# Penicillin allergy delabeling in long-term care facilities: if not now, then when?

**DOI:** 10.1017/ash.2025.30

**Published:** 2025-02-24

**Authors:** Kap Sum Foong, Shira Doron, Alysse Wurcel

**Affiliations:** 1 Department of Medicine, Division of Geographic Medicine and Infectious Diseases, Tufts Medical Center, Boston, MA, USA; 2 Section of Infectious Diseases, Boston Medical Center, Boston, MA, USA

## Current state and gaps in penicillin allergy management in long-term care settings

Approximately 10% of the United States (US) population has a penicillin allergy label (PAL), yet studies show that up to 90% of these individuals are not truly allergic and can safely receive penicillin.^1–5^ This mislabeling contributes to negative health outcomes, including higher use of broad-spectrum antibiotics, increased healthcare costs, and greater risks of adverse effects such as *Clostridioides difficile* infection (CDI) and antimicrobial resistance.^6–10^

Efforts to remove inaccurate PALs—known as delabeling—have expanded over the past decade.^4,11,12^ Historically performed by allergists in clinics, structured delabeling programs have been increasingly implemented across diverse clinical settings, including ambulatory clinics, emergency departments, general medicine wards, intensive care units, surgical wards, and inpatient rehabilitation facilities.^12–18^ These programs often utilize comprehensive allergy history assessments, risk stratification tools like PEN-FAST, and direct oral drug challenges.^1,4,12,19^ For patients with low-risk allergy histories, delabeling may be performed based on history alone or through oral amoxicillin challenges, whereas those with high-risk histories may require penicillin skin testing followed by oral amoxicillin challenges, or referral to an allergist for further evaluation.^1,4,12,20^ Programs led by non-allergist healthcare providers, including pharmacists and infectious disease specialists, have demonstrated success across settings.^12,21–23^

Despite these advancements, structured penicillin allergy evaluation and delabeling programs remain limited in long-term care (LTC) settings.^12^ Consequently, LTC residents with PALs continue to face barriers to optimal antibiotic therapy, placing them at a potentially increased risk for negative health outcomes. Studies report that nearly one in four LTC residents carries a PAL, and these residents are less likely to receive beta-lactam antibiotics, potentially leading to increased use of broad-spectrum antibiotics or antibiotics at higher risk for CDI such as fluoroquinolones.^24–26^

A recent call to action highlights the need to include underrepresented research participants to develop and evaluate the impact of interventions on health outcomes in heterogeneous populations.^27^ These principles extend to other populations marginalized beyond sex, race, and ethnicity, including older adults in LTC settings, who are frequently overlooked in research. LTC residents, already at greater risk for infections and adverse outcomes from broader-spectrum antibiotic use, face setting-specific barriers that can exacerbate existing health inequities.^28,29^

## Unique challenges in long-term care settings

Implementing penicillin allergy delabeling programs in LTC settings presents distinct challenges rooted in both structural and operational constraints. (Table 1) Staffing shortages, high turnover, burnout, and limited resources, exacerbated during the COVID-19 pandemic, create significant barriers to initiatives such as comprehensive penicillin allergy assessment and delabeling protocols.^44–46^

**Table 1.** Challenges and potential solutions for implementing penicillin allergy delabeling in long-term care settings

Challenges	Potential Solutions
**Staffing shortages, high turnover, burnout, and limited resources**	**Standardize protocols** using **risk stratification** tools like PEN-FAST to streamline delabeling with minimal resource demand. Advocate for **reimbursement** to offset costs.^1,9,30–34^
**Research gaps**	**Advocating for funding** to generate evidence on the feasibility, sustainability, and outcomes of delabeling programs in LTC settings. **Expand research scope** to include cost-effectiveness, scalability, and the role of geriatric-focused interventions. **Publish findings in geriatric literature.**
**Regulatory requirements and documentation burden**	Develop **streamlined documentation** protocols to simplify regulatory compliance while maintaining evidence-based practices.^1,30^
**Lack of access to allergy specialists**	Use **telemedicine** to connect LTC facilities with allergists for remote supervision and consultation.^35^
**Lack of education and awareness among LTC healthcare providers**	Provide **comprehensive training** through webinars, online courses, and practical tools to enhance LTC providers’ confidence and competence.^36–42^
**Complex resident care, including cognitive impairment and polypharmacy**	Engage families and caregivers through education **and shared decision-making** to support delabeling in residents with cognitive impairment or complex medical histories.^41,43^

LTC, long-term care.

Another critical gap lies in the limited research on penicillin allergy delabeling in LTC settings. While recent studies have examined the prevalence of PALs and barriers to delabeling, evidence on the implementation of such programs in LTC remains scarce.^24–26,30^ Although one small study demonstrated the feasibility of a penicillin allergy delabeling program in a post-acute rehabilitation facility, its cost-effectiveness, sustainability, and impact were not evaluated.^18^ This research gap hinders the development and implementation of tailored, evidence-based delabeling strategies to optimize antibiotic prescribing practices in LTC populations.

Regulatory requirements also hinder penicillin allergy delabeling efforts.^47^ LTC facilities face stringent oversight and burdensome documentation mandates, which can discourage additional interventions aimed at addressing inaccurate PALs. Concerns about regulatory scrutiny and liability further exacerbate hesitancy among healthcare providers when managing allergies in vulnerable LTC populations.^30^

The national shortage of allergists, combined with insufficient education and awareness among LTC healthcare providers further complicate these implementation efforts.^30,48^ LTC residents, particularly in rural settings, often lack access to specialist care including allergists, leaving LTC clinicians without the resources or confidence to manage penicillin allergy delabeling safely.^49^ Many LTC healthcare providers are unfamiliar with the evidence supporting penicillin allergy delabeling, and misconceptions about penicillin allergies and the perceived risks of using beta-lactam antibiotics in LTC residents with PALs can contribute to overly cautious antibiotic prescribing practices.^30^

Additional challenges arise from the complexity of resident care.^50^ Cognitive impairment and dementia, prevalent among LTC residents, may interfere with accurate reporting of allergic reactions.^30,51^

## Potential solutions and call to action

Proposed solutions can be categorized into short-term and long-term goals. Short-term goals focus on strategies that are easily integrated into individual LTC healthcare provider workflows or facility-level practices. These include enhancing education, training, and counseling for LTC healthcare providers, residents, and families about the benefits of penicillin allergy delabeling while dispelling misconceptions about PALs.^30,52^ Free resources such as webinars, online courses, and educational videos can build provider confidence.^36,37,52^ Additionally, family education and shared decision-making, proven effective in pediatric settings, can improve caregivers’ understanding of the penicillin allergy evaluation and delabeling process, supporting informed decision-making for LTC residents with cognitive impairment or dementia.^53^ LTC healthcare providers can also utilize existing guidelines and resources on beta-lactam cross-reactivity risks to make more informed antibiotic choices.^4,54^

Long-term goals require systems-level changes to address structural and operational barriers effectively. Developing streamlined, standardized protocols and tools is critical to promoting consistent and effective penicillin allergy delabeling in LTC settings.^12,20^ Risk stratification tools, such as PEN-FAST, offer low-resource, evidence-based solutions for identifying residents with low-risk allergy history suitable for penicillin allergy delabeling.^19^ These tools can be integrated into routine care through protocols that define clear criteria for verifying PAL, outline step-by-step procedures for conducting direct oral challenges, and provide guidelines for documenting outcomes.^30,55^ Additionally, expanding access to telemedicine for remote allergy consultations offers a cost-effective way to connect LTC facilities with allergists for supervision of allergy testing.^56,57^ For example, the use of telemedicine during the COVID-19 pandemic demonstrated the feasibility of penicillin allergy delabeling in other settings.^35^

Addressing the two distinct LTC populations—short-stay and long-stay residents—adds complexity. Short-stay residents require continuity of delabeling into outpatient settings, while long-stay residents necessitate reliable documentation within electronic medical records. These factors underline the need for detailed, setting-specific approaches rather than generic solutions.

Targeted funding for research from agencies (eg, Centers for Disease Control and Prevention, Agency for Healthcare Research and Quality, and Centers for Medicare & Medicaid Services) is critical to advancing penicillin allergy delabeling in LTC settings. Research can support the development of tailored approaches and scalable models to improve implementation. Greater engagement in geriatric-focused research and practice is essential to bridge knowledge gaps. Publishing in geriatric journals, presenting at relevant conferences, and collaborating with organizations like the American Geriatrics Society can promote the adoption of evidence-based strategies. Geriatricians, with their close connections to LTC residents and families, are key advocates for integrating these practices. Advocacy for supportive policies is equally important. Policymakers should incorporate penicillin allergy delabeling into antibiotic stewardship programs, with reimbursement mechanisms to offset costs and ensure feasibility. National initiatives like the Penicillin Allergy Verification and Evaluation Act could provide scalable models for systematic implementation.^31^

## Conclusion

Penicillin allergy delabeling in LTC settings is a critical component of improving antibiotic stewardship and ensuring equitable access to effective antibiotic treatment. However, without targeted research funding and tailored implementation strategies, these efforts risk imposing additional burdens on already strained LTC systems. Prioritizing health equity and actionable policy solutions is essential to addressing these challenges effectively.
